# ATF6alpha Promotes Astroglial Activation and Neuronal Survival in a Chronic Mouse Model of Parkinson’s Disease

**DOI:** 10.1371/journal.pone.0047950

**Published:** 2012-10-24

**Authors:** Koji Hashida, Yasuko Kitao, Hirofumi Sudo, Yoshitaka Awa, Shinichiro Maeda, Kazutoshi Mori, Ryosuke Takahashi, Munekazu Iinuma, Osamu Hori

**Affiliations:** 1 Department of Neuroanatomy, Kanazawa University Graduate School of Medical Sciences, Kanazawa, Ishikawa, Japan; 2 Core Research for Evolutional Science and Technology (CREST), Japan Science and Technology (JST), Tokyo, Japan; 3 Department of Biophysics, Graduate School of Science, Kyoto University, Kyoto, Japan; 4 Department of Neurology, Graduate School of Medicine, Kyoto University, Kyoto, Japan; 5 Laboratory of Pharmacognosy, Gifu Pharmaceutical University, Gifu City, Gifu, Japan; Okayama University Graduate School of Medicine, Dentistry and Pharmaceutical Sciences, Japan

## Abstract

Accumulating evidence suggests a crucial role for the unfolded protein response (UPR) in Parkinson’s disease (PD). In this study, we investigated the relevance of the UPR in a mouse model of chronic MPTP/probenecid (MPTP/P) injection, which causes severe and persistent degeneration of dopaminergic neurons. Enhanced activation of the UPR branches, including ATF6α and PERK/eIF2α/ATF4, was observed after MPTP/P injections into mice. Deletion of the ATF6α gene accelerated neuronal degeneration and ubiquitin accumulation relatively early in the MPTP/P injection course. Surprisingly, astroglial activation was strongly suppressed, and production of the brain-derived neurotrophic factor (BDNF) and anti-oxidative genes, such as heme oxygenase-1 (HO-1) and xCT, in astrocytes were reduced in ATF6α −/− mice after MPTP/P injections. Decreased BDNF expression in ATF6α −/− mice was associated with decreased expression of GRP78, an ATF6α-dependent molecular chaperone in the ER. Decreased HO-1 and xCT levels were associated with decreased expression of the ATF4-dependent pro-apoptotic gene CHOP. Consistent with these results, administration of the UPR-activating reagent tangeretin (5,6,7,8,4′-pentamethoxyflavone; IN19) into mice enhanced the expression of UPR-target genes in both dopaminergic neurons and astrocytes, and promoted neuronal survival after MPTP/P injections. These results suggest that the UPR is activated in a mouse model of chronic MPTP/P injection, and contributes to the survival of nigrostriatal dopaminergic neurons, in part, through activated astrocytes.

## Introduction

Parkinson’s disease (PD) is a progressive neurodegenerative disease pathologically characterized by the selective loss of nigrostriatal dopaminergic neurons and the presence of protein aggregates, known as Lewy bodies [1]. Although the etiology of PD is not fully understood, several genetic and environmental factors have been discovered that are utilized to model PD in experimental animals [2]. 1-Methyl-4-phenyl-1,2,3,6-tetrahydropyridine (MPTP), an inhibitor of mitochondrial complex I, induced human Parkinsonism [3], and has, therefore, been widely used to generate a PD model in animals. MPTP is highly lipophilic and crosses the blood-brain barrier. In the brain, it is converted to the active form, 1-methyl-4-phenylpyridinium (MPP^+^) by astrocytes, and is taken up by dopaminergic neurons through the dopamine transporter (DAT). Accumulating evidence suggests that, compared with acute and subacute MPTP injection, chronic administration of MPTP with probenecid (MPTP/P) into mice causes more severe neurodegeneration and dopamine (DA) depletion that resembles human PD [4], [5].

Although MPTP causes oxidative stress and energy depletion because of impaired mitochondrial function, recent studies suggest that MPTP also causes endoplasmic reticulum (ER) stress, a type of intracellular stress that is characterized by the accumulation of unfolded proteins in the ER. ER stress occurs when cells are in conditions such as glucose starvation (energy depletion), oxygen deprivation, protein modification inhibition, and disturbance of Ca^2+^ homeostasis. Eukaryotic cells respond to ER stress by activating a set of pathways known as the unfolded protein response (UPR) [6]. In mammals, the UPR is transmitted through 3 types of sensor proteins; double-stranded RNA-activated protein kinase (PKR)–like ER kinase (PERK), inositol-requiring enzyme 1α (Ire1α), and activating transcription factor 6α (ATF6α) [6]. Ire1α and ATF6α downstream genes include molecular chaperones in the ER, such as glucose-regulated protein78 (GRP78), and oxygen-regulated protein 150 (ORP150), and ER-associated degradation (ERAD) molecules such as Derlins, ER degradation enhancing alpha-mannosidase-like protein (EDEM), and homocysteine-inducible endoplasmic reticulum stress protein (Herp). In contrast, PERK downstream genes include eukaryotic translation initiation factor 2 (eIF2α), which suppresses general protein synthesis to reduce protein loads into the ER, and activating transcription factor 4 (ATF4), which upregulates the expression of anti-oxidative genes such as heme oxygenase 1 (HO-1) and cystine/glutamate antiporter (xCT). PERK also upregulates the pro-apoptotic transcriptional factor C/EBP homologous protein (CHOP) [7]. Cell culture models [8], [9] and the acute MPTP injection models [10], [11] demonstrated the UPR has important roles in promoting neuronal survival against MPTP neurotoxicity. Furthermore, a recent report demonstrated that MPP^+^-associated oxidative stress enhanced the interaction between phosphorylated p38 mitogen-activated protein kinase (p38MAPK) and ATF6α, causing increased transcriptional activity of ATF6α [12]. These findings suggest an important communication between the oxidative stress response and the UPR in PD pathogenesis.

We initiated this study by estimating the UPR activation status after chronic MPTP/P injections into wild-type mice. We compared neurodegeneration, protein aggregation, and glial activation levels between wild-type and ATF6α −/− mice. Finally, we estimated the neuroprotective property of the UPR-activating compound, tangeretin (5,6,7,8,4′-pentamethoxyflavone; IN19), in the mouse model of chronic MPTP/P injection.

## Materials and Methods

### Ethics Statement

All animal care and handling procedures were approved by the Animal Care and Use Committee of Kanazawa University (No. 71241-1).

### Materials

MPTP and Cremophore EL were purchased from Sigma (St Louis, MO). Probenecid and dimethy sulfoxide (DMSO) were purchased from Wako Chemicals (Osaka, Japan). The UPR-activating compound tangeretin (IN19) was isolated as described previously [13].

### Mice and Chronic MPTP/P Injection PD Model

ATF6α −/− mice were generated as described previously [14], and backcrossed to the C57BL/6 strain more than 8 times. Wild-type and ATF6α −/− male mice (aged 12–15 weeks and weighing 26–30 g) were used for the experiments. The chronic MPTP/P injection PD model was created as described previously with some modifications. In brief, mice were administered MPTP (20 mg/kg in saline, subcutaneously) and probenecid (250 mg/kg in DMSO, intraperitoneally) twice a week for 5 weeks [4]. At the indicated times, brain samples were prepared for histological analyses, RT-PCR/quantitative real time RT-PCR (qRT-PCR) and Western blotting as described. In some experiments, mice were administered tangeretin (10 mg/kg, per oral, in saline including 10% Cremophore EL and 10% DMSO) or the dissolving solution (vehicle) 24 h and 2 h before MPTP/P injections [11].

### RT-PCR and Quantitative Real Time RT-PCR (qRT-PCR)

Total RNA was extracted from the ventral midbrain or caudate putamen (CPu) of each mouse using RNAzol®RT (Molecular Research Center Inc, Cincinnati, OH). RT reactions containing 1 µg of total RNA were performed using PrimeScript (Takara, Shiga, Japan). The individual cDNA species were amplified in a reaction mixture containing 1 unit of Taq DNA polymerase (Takara) and specific primers for ATF6, GRP78, ORP150, ATF4, HO-1, CHOP, X-box binding protein 1 (XBP-1), Sec61β, GFAP, Iba1, andβ-actin as described previously. In some experiments, cDNA derived from cultured astrocytes treated with tunicamycin for 8 h [15] was used as a positive control for XBP1 activation. For qRT-PCR, cDNA was amplified with THUNDERBIRD™ SYBR qPCR® Mix (TOYOBO CO, LTD, Osaka, Japan) by using specific primers for brain-derived neurotrophic factor (BDNF), leukemia inhibitory factor (LIF), interleukin-6 (IL-6), GRP78, ORP150, CHOP, xCT, manganese superoxide dismutase (MnSOD), and β-actin. The comparative Ct method was used for data analyses with MxPro 4.10 (Agilent Technologies, Santa Clara, CA). Values for each gene were normalized to β-actin expression levels.

### Western Blotting and ELISA

Brain samples from the ventral midbrain or CPu were solubilized in buffer containing 1% NP40, 0.1% SDS, and 0.2% deoxycholate, and subjected to Western blotting with following antibodies: BDNF (Epitomics, Burlingame, CA), GRP78 (StressGen, Victoria, British Columbia, Canada), HO-1 (Abcam, Cambridge, UK), xCT (Thermo Scientific, Rockford, IL), GLT-1 (Millipore, Temecula, CA), LIF (Santa Cruz Biotechnology, Santa Cruz, CA), GFAP (Dako, Glostrup, Denmark) and β-actin (Sigma). Primary antibody binding was visualized using alkaline phosphatase-conjugated secondary antibodies or the ECL system (GE Healthcare Bio-Sciences Corp., Piscataway, NJ). IL-6 levels in the brain samples were measured using ELISA (eBioscience, San Diego CA).

### Histological and Immunohistochemical Analyses

Brains were removed from mice after perfusion with 4% paraformaldehyde, and postfixed in the same fixative for 4 hours at 4°C. After cryoprotected in 30% sucrose, brains were cut in serial coronal 10 µm-thick sections containing the CPu (from Bregma+1.34 mm to Bregma+0.26 mm) and the midbrain covering the whole SNpc (from Bregma-2.80 mm to Bregma-3.80 mm) on a cryostat, and mounted in series on ten slides (around ten sections were mounted on each slide). One out of these ten slides, representing a set of sections 100 µm apart, were processed for immunohistochemistry, and the negative control, in which the primary antibody was omitted, was performed in parallel with each procedure. Primary antibodies used were; anti-TH (Sigma), anti-GRP78, anti-HO-1, anti-GLT-1, anti-Ubiquitin (StressGen), anti-cleaved caspase 3 (Cell Signaling Technology, Danvers, MA), anti-BDNF, anti-GFAP, anti-Iba1. In some cases, the cell nucleus was visualized with DAPI (Sigma), and Cresyl violet (Sigma) was used for counterstaining. Appropriate Alexa Fluor 488, Cy3-conjugated IgG or peroxidase-conjugated IgG was used as a secondary antibody. Confocal images were obtained by using Nikkon EZ-C1. In the process of apoptosis, cleaved caspase 3 was observed both in the cytosol and nucleus. Expression of GRP78, ORP150, HO-1, and BDNF was immunohistochemically detected mainly in the cell body of neurons and/or astrocytes, and that of GLT-1 was detected in the process of astrocytes.

### Image Quantification

Quantification of the RT-PCR, Western Blotting, and immunohistochemical analyses were performed using Image J (version 1.42, Wayne Rasband, National Institutes of Health). The number of TH positive neurons in the SNpc was counted in five representative sections out of ten sections mounted on one slide, which covered the whole SNpc. Statistical analyses were performed using Bonferroni/Dunn test following a one-way ANOVA.

**Figure 1:**
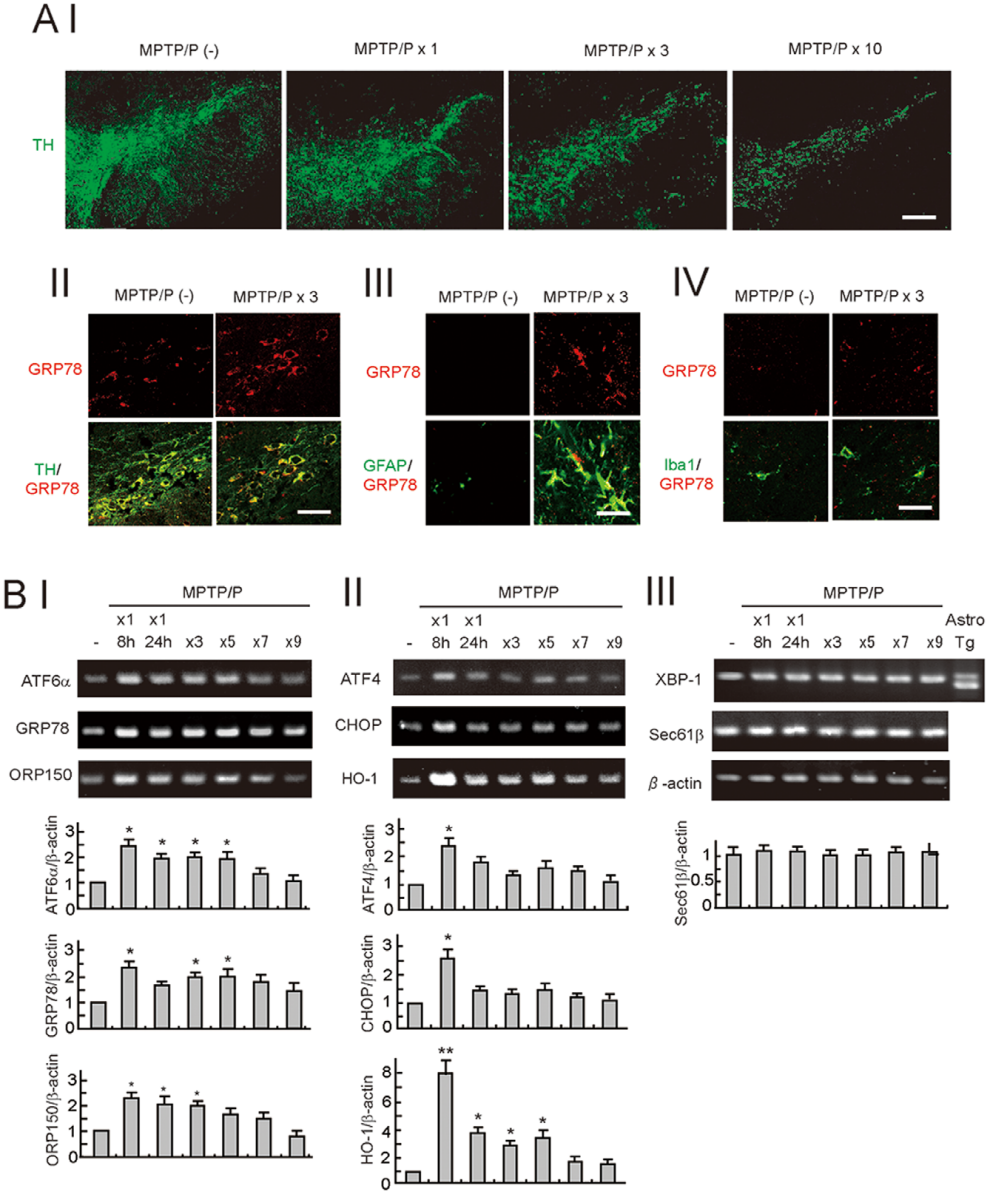
The unfolded protein response (UPR) in a mouse model of chronic MPTP/P injection. A, Neurodegeneration (I) and UPR activation (II, III, IV) in the SNpc after MPTP/P injections. Brain sections, including the SN from wild-type mice injected with or without MPTP/P were immunostained with the TH, GRP78, GFAP, and Iba1 antibodies. Scale bars = 50 µm (I), 30 µm (II), 20 µm (III), 20 µm (IV). B, Gene expression in the UPR branches after MPTP/P injections. Total RNA (1 µg) isolated from the ventral midbrain of mice was subjected to RT-PCR with specific primers for ATF6α-target genes (I), ATF4-target genes (II), XBP1-target genes, and β-actin (III). The far right lane in (III) indicates the unspliced and spliced form of the XBP1 from cultured astrocytes treated with thapsigargin (an ER stressor). The relative intensity of the bands derived from the mice without MPTP/P injection is designated as one. Values shown are the mean ± S.D. *P<0.05, **P<0.01 compared with mice without MPTP/P administration (n = 4).

**Figure 2:**
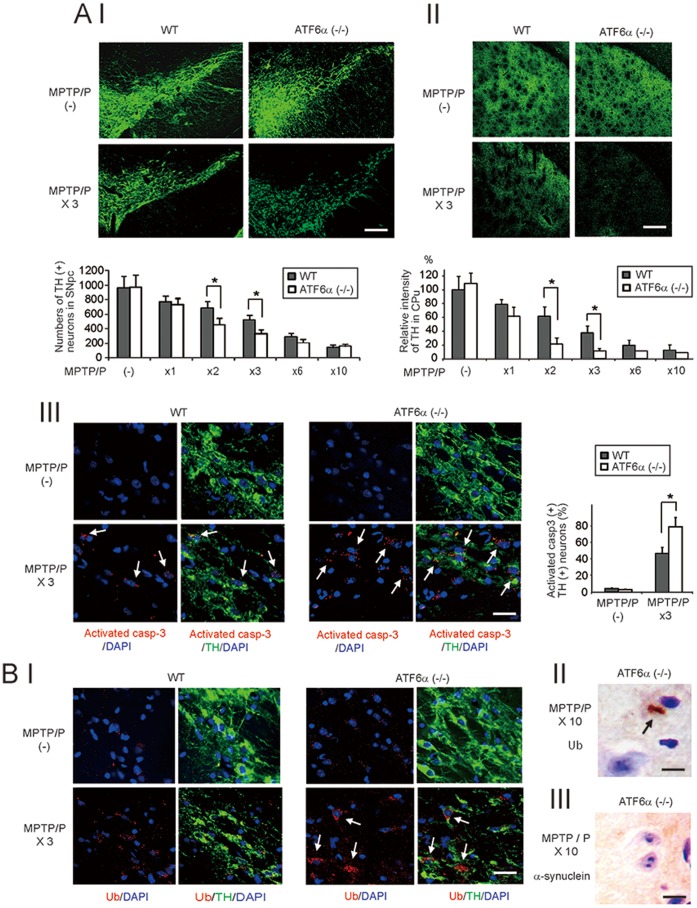
Accelerated neurodegeneration and Ub accumulation in ATF6α −/− mice after MPTP/P injection. A, TH immunereactivity (I, II) and activated caspase 3 (III) after MPTP/P injections. Brain sections, including the SN (I, III) or CPu (II), from wild-type and ATF6α −/− mice that were injected with or without MPTP/P were immunostained with TH and activated caspase 3 antibodies. The number of TH-positive neurons in the SNpc (I) and TH or activated caspase3 intensity in the CPu (II) are shown in the graph. In III, the nuclei are stained with DAPI. Arrows indicate activated caspase 3-positive, TH-positive neurons. The relative number of activated caspase 3-positive, TH-positive neurons in the SNpc are also shown in the graph. Values shown are the mean ± S.D. Scale bars = 50 µm (I), 100 µm (II), 30 µm (III). *P<0.05, compared between wild-type and ATF6α −/− mice (n = 4). B, Ub accumulation (I) and intracellular inclusion bodies (II) in ATF6α −/− mice after MPTP/P injections. (I) Brain sections, including the SN, from mice that were injected with or without MPTP/P were immunostainined with the TH and Ub antibodies. Nuclei were stained with DAPI. Note that in ATF6α −/− mice, Ub accumulation was observed in cells with reduced TH immunoreactivity (arrows). (II, III) Brain sections, including the SN, from ATF6α −/− mice that were injected 10 times with MPTP/P were immunostained with the Ub (II) or α-synuclein antibody, followed by counterstaining with cresyl violet. Arrows indicate Ub-positive inclusion bodies. Scale bars = 30 µm (I), 20 µm (II, III).

## Results

### UPR in the Chronic MPTP/P Injection PD Model

When MPTP/P was injected into C57BL/6 wild-type mice, the number of TH-positive dopaminergic neurons in the SNpc and the intensity of TH in the CPu gradually decreased (Fig. 1 A I, Fig. 2 A I, II). This result indicates degeneration of the nigrostriatal dopaminergic neurons in this model. Further immunohistochemical studies revealed increased expression of the UPR-target gene GRP78 after MPTP/P injections in TH-positive dopaminergic neurons (Fig. 1 A II) and GFAP-positive activated astrocytes (Fig. 1 A III), but not in Iba1-positive microglia (Fig. 1 A IV). To evaluate the activation status of the 3 major UPR pathways after MPTP/P injections, we analyzed the expressions of genes involved in each pathway by using RT-PCR (Fig.1 B I, II, III). The transcripts of ATF6α and its downstream genes, such as GRP78 and ORP150, were enhanced 2.5, 2.3, and 2.4 fold, respectively, 8 h after the first MPTP/P injection. The increased expression tended to last until at least fifth MPTP/P injection (Fig. 1 B I). Transcripts of ATF4 and its downstream genes, such as CHOP and HO-1, which is also a target of NF-E2-related factor 2 (Nrf2), were enhanced 2.3, 2.5, and 7.6 fold, respectively, 8 h after the first MPTP/P injection. However, the expression levels dropped to 1.7, 1.4, and 3.9 fold, respectively, 24 h after the first injection (Fig. 1 B II). In contrast, the Ire1/XBP-1 pathway, and XBP-1, which can be detected by the altered splicing pattern [6], was not activated. In addition, the downstream gene Sec61β was not upregulated after MPTP/P injection (Fig. 1 B III).

### Accelerated Neurodegeneration and Ub Accumulation in ATF6α −/− Mice after MPTP/P Injections

To evaluate the neuroprotective role of the UPR in the chronic MPTP/P injection model, we immunehistochemically compared nigrostriatal neuronal degeneration between wild-type and ATF6α −/− mice (Fig. 2 A I, II). In the control condition (without MPTP/P administration), the number of TH-positive neurons in the SNpc and the intensity of TH in the CPu were not significantly different among the wild-type and ATF6α-deficient mice. In contrast, in the early course of MPTP/P injections (2^nd^ and 3^rd^ injections), the number of TH-positive neurons in the SNpc and the intensity of TH in the CPu were significantly lower in ATF6α −/− mice compared to wild-type mice. Consistent with these results, higher numbers of activated caspase-3-positive, TH-positive neurons were observed in ATF6α −/− mice (74%) compared to wild-type mice (47%; Fig. 2 A III). The specificity of the antibody and the appropriate immunoreactivity of the antigen were confirmed by the negative control experiment where primary antibody was omitted (Fig. S 2 A) and the serial photograph of the confocal images (Fig. S 2 B), respectively. In the later injections (6^th^–10^th^ injections), however, the nigrostriatal dopaminergic neurons had degenerated to similar levels in both cohorts (Fig.2 A I, II). Egawa et al. recently demonstrated the presence of Ub-positive inclusions in ATF6α −/− mice after acute MPTP injection [12]. Therefore, we assessed Ub accumulation in our model. In the control condition, slight Ub immunoreactivity in the cell body was observed in TH-positive neurons of both cohorts (Fig. 2 B I, upper row). However, after the 3^rd^ MPTP/P injection, Ub accumulation was observed in the degenerating dopaminergic neurons in the ATF6α −/− SNpc, but not in the wild-type SNpc (Fig. 2 B I, arrows). In 29% (4 of 14) of ATF6α −/− mice, Ub-positive inclusions were observed in the neurons after 10^th^ MPTP/P injection (Fig. 2 B II), but not in wild-type neurons (data not shown). These inclusions were negative or very weak for α-synuclein immunoreactivity (Fig. 2 B III), as previously described [12].

**Figure 3:**
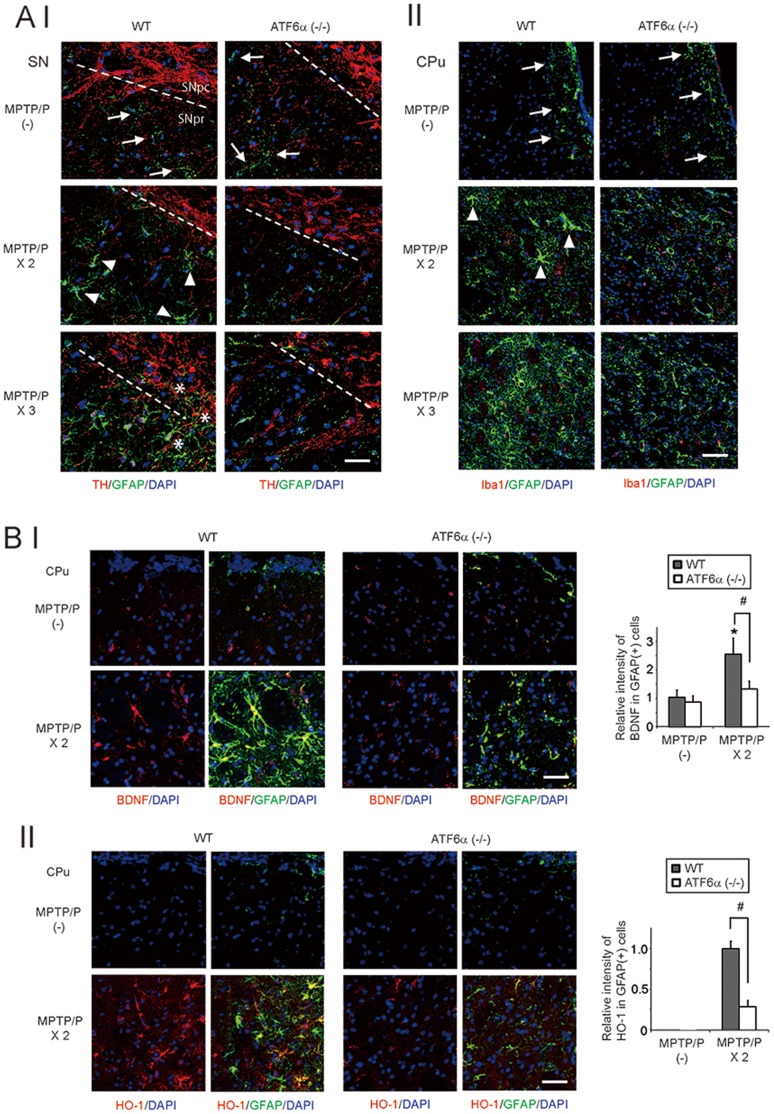
Impaired astroglial activation in ATF6α−/− mice after MPTP/P injections. A, GFAP, TH, and Iba1 expression after MPTP/P injections. Brain sections, including the SN (I) or CPu, (II) from wild-type and ATF6α−/− mice that were injected with or without MPTP/P were immunostained with GFAP, TH, and Iba1 antibodies. Nuclei were stained with DAPI. Arrows and arrowheads indicate non-activated and activated astrocytes, respectively. Asterisks indicate activated astrocytes penetrating into the SNpc. Scale bars = 30 µm. B, BDNF (I) and HO-1 (II) expression in astrocytes after MPTP/P injections. Brain sections, including the CPu, from wild-type and ATF6α −/− mice that were injected with or without MPTP/P were immunostained with the BDNF, HO-1, and GFAP antibodies. Nuclei were stained with DAPI. The relative intensity of BDNF (I) or HO-1 (II) in the GFAP-positive cells is shown in the graph. The intensity of the signals derived from wild-type mice without MPTP/P injection is designated as one. Values shown are the mean ± S.D. *P<0.05, compared between wild-type and ATF6α−/− mice (n = 4). Scale bars = 30 µm.

**Figure 4:**
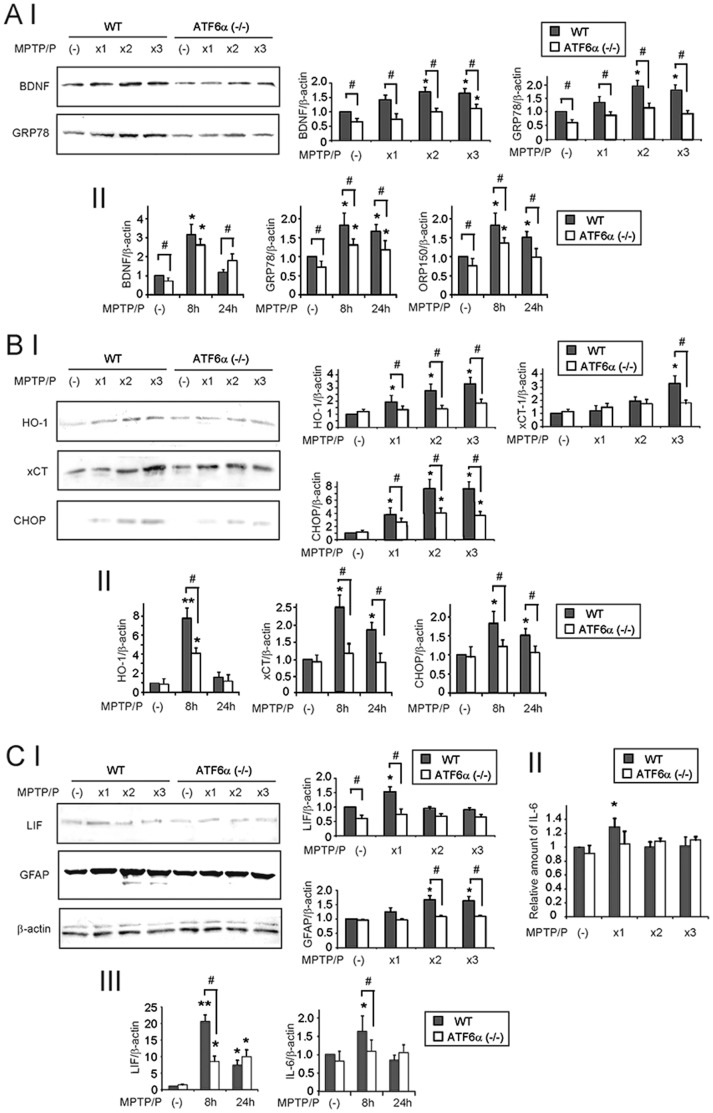
Reduced UPR levels and gene expression in ATF6α−/− mice after MPTP/P injections. Protein expression of neurotrophic factor (A I), anti-oxidative genes (B I), astrogliosis-inducing factor (C I, II), and the UPR-target genes (A I, B I). Protein extracts from brains (CPu) of wild-type or ATF6α −/− mice that were injected or not injected with MPTP/P were subjected to Western blotting with the indicated antibodies (A I, B I, C I), or subjected to IL-6 ELISA (C II). Relative intensities are shown in the graphs. The intensity of the genes from wild-type mice without MPTP/P administration is designated as one. Values shown are the mean ± S.D. *P<0.05, compared with mice without MPTP/P administration. ^#^P<0.05, compared between wild-type and ATF6α −/− brains (n = 4). Transcripts of neurotrophic factor (A II), anti-oxidative genes (B II), astrogliosis-inducing factors (C III), and the UPR-target genes (A II, B II). Total RNA (1µg) isolated from wild-type or ATF6α −/− brains (CPu) at indicated times after 1^st^ MPTP/P injection was subjected to qRT-PCR with specific primers for the indicated genes. The relative intensity of the genes from wild-type mice not administered MPTP/P is designated as one. Values shown are the mean ± S.D. *P<0.05, **P<0.01, compared with the mice without MPTP/P administration. ^#^P<0.05, compared between wild-type and ATF6α −/− brains (n = 4).

### Impaired Astroglial Activation in ATF6α −/− mice after MPTP/P Injections

Accelerated neurodegeneration was observed in ATF6α −/− mice predominantly when glial cells were activated (Fig. S1). Therefore, glial activation was assessed in wild-type and ATF6α −/− mice. Immunohistochemical analyses in the SN and CPu revealed that in the control condition, GFAP-positive astrocytes were sparsely observed in both mouse cohorts (Fig. 3 A I, II, arrows). However, in the period from the 2^nd^ to 3^rd^ MPTP/P injection, the features of astroglial activation (enlarged cell bodies and thick processes) in the SN and CPu were observed more frequently in wild-type mice compared to ATF6α −/− mice (Fig. 3 A I, II, 2^nd^ and 3^rd^ rows). In the wild-type SN, astrocytes became enlarged in the SN pars reticulata (SNpr) first (arrowheads), and then penetrated into the SNpc (asterisks), but ATF6α −/− astrocytes were not enlarged after MPTP/P injections. In the CPu, wild-type astrocytes near the lateral ventricle (arrows) and corpus callosum (data not shown) became enlarged and, almost simultaneously, spread over the CPu, but again, ATF6α −/− astrocytes were not enlarged after MPTP/P injections. Consistent with the immunohistochemical observations, Western blot analyses revealed enhanced GFAP expression in wild-type mice, but not in ATF6α −/− mice, after the 2^nd^ and 3^rd^ MPTP/P injections (Fig.4 C I). In contrast to high levels of astroglial activation, microglial activation was modest in this model, and the differences in the microglia morphology were not clear between wild-type and ATF6α −/− mice after the 2^nd^ MPTP/P injection (Fig. 3 A II).

Activated astrocytes contribute to neuroprotection in several ways, including neurotrophic factor synthesis, enhancement of anti-oxidative systems, and glutamate metabolism [16], [17]. Therefore, we compared the expression of BDNF (a neurotrophic factor), HO-1 (an anti-oxidative gene), and GLT-1 (a glutamate transporter) in wild-type and ATF6α −/− mice. Immunohistochemical analyses revealed that BDNF and HO-1 expression (Fig. 3 B I, II), but not GLT-1 expression (Fig. S2 C), were higher after MPTP/P injections in wild-type astrocytes compared with ATF6α −/− astrocytes in the CPu.

**Figure 5:**
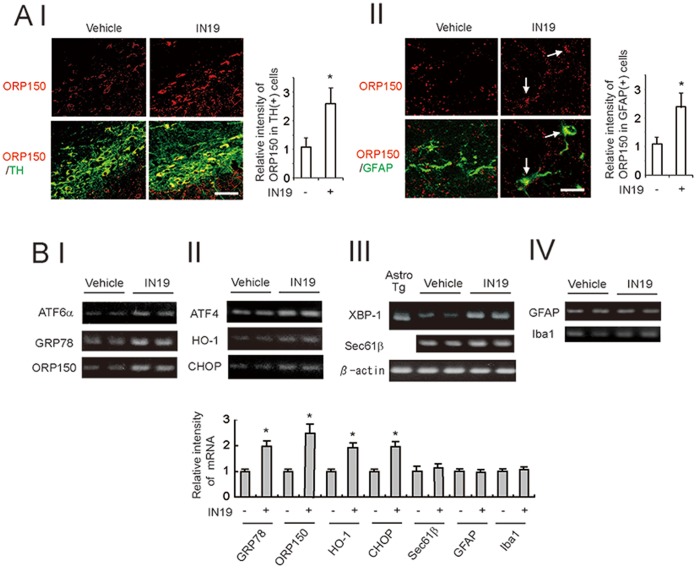
UPR in the brain after tangeretin (IN19) administration. A, UPR activation in dopaminergic neurons (I) and astrocytes (II) by IN19. Brain sections, including SN from wild-type mice administered or not administered IN19 for 2 weeks (4 times/week) were immunostained with the ORP150, TH, and GFAP antibodies. The relative intensity of ORP150 in the TH-positive cells (I) or the GFAP-positive cells (II) is shown in the graph. The intensity of the signals derived from vehicle-administered mice is designated as one. Values shown are the mean ± S.D. *P<0.05, compared between vehicle- and IN19-administered mice (n = 4). Scale bars = 30 µm (I), 20 µm (II). B, Gene expression in the UPR branches and gliosis after IN19 administration. Total RNA (1 µg) isolated from brain samples, including the ventral midbrain, with or without IN19 administration was subjected to RT-PCR with specific primers for ATF6α-related genes (I), ATF4-related genes (II), XBP1-related genes and β-actin (III), gliosis-related genes (IV). The far left lane in (III) indicates the unspliced and spliced form of the XBP1 from cultured astrocytes treated with thapsigargin (an ER stressor). Note that XBP-1 transcripts were upregulated by IN19 administration, but they were not activated. The relative intensity of the bands derived from mice without IN19 administration is designated as one. Values shown are the mean ± S.D. *P<0.05, **P<0.01 compared with mice not administered MPTP/P (n = 4).

**Figure 6:**
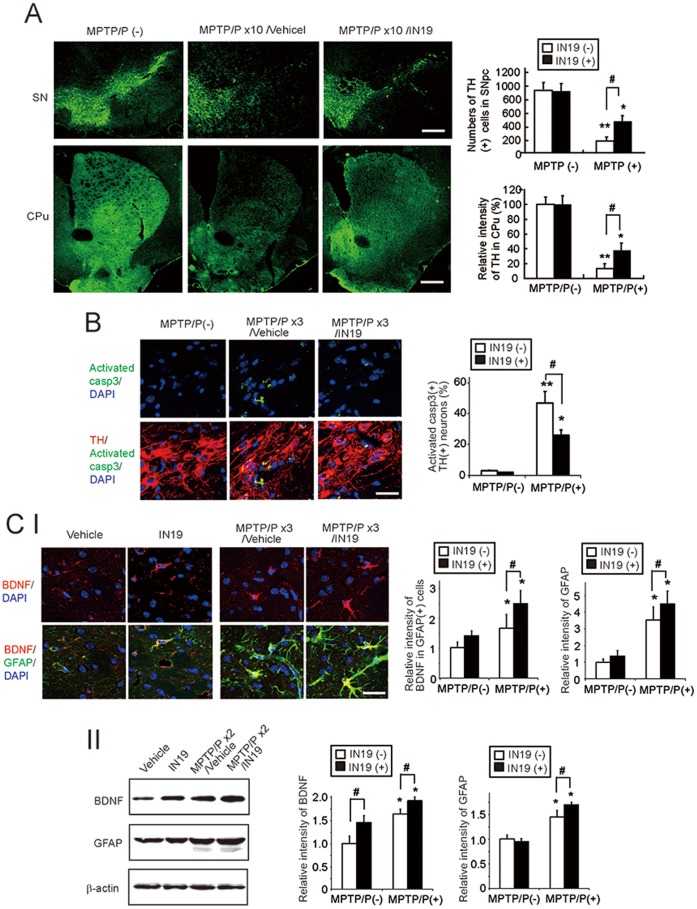
IN19-mediated neuroprotection in a chronic MPTP/P injection model. A, The effect of IN19 on neurodegeneration after MPTP/P injections. Brain sections, including the SN (upper row) or CPu (lower row), from wild-type mice that were injected or not injected with MPTP/P, in the presence or absence of IN19, were immunostained with the TH antibody. The number of TH-positive neurons in the SNpc and TH intensity in the CPu are shown in the graph. Values shown are the mean ± S.D. *P<0.05, **P<0.01, compared with mice without MPTP/P administration. ^#^P<0.05, compared between vehicle- and IN19-administered mice (n = 4). Scale bars = 100 µm (SN), 200 µm (CPu). B, The effect of IN19 on caspase-3 activation after MPTP/P injections. Brain sections, including the SN from wild-type and ATF6α −/− mice that were injected with MPTP/P, in the presence or absence of IN19, were immunostained with the activated caspase 3 and TH antibodies. Nuclei were stained with DAPI. The relative number of activated caspase 3-positive, TH-positive neurons are shown in the graph. Values shown are the mean ± S.D. *P<0.05, **P<0.01, compared with mice without MPTP/P administration. ^#^P<0.05, compared between vehicle- and IN19-administered mice (n = 4). Scale bar = 30 µm. C, The effect of IN19 on BDNF expression. (I) Brain sections, including the CPu, from wild-type mice that were injected or not injected with MPTP/P, in the presence or absence of IN19, were immunostained with the BDNF and GFAP antibodies. Nuclei were stained with DAPI. The relative intensity of BDNF or GFAP is shown in the graph. The intensity of the signals derived from vehicle-administered, not MPTP/P-injected mice, is designated as one. Values shown are the mean ± S.D. *P<0.05, compared with mice without MPTP/P administration,^ #^P<0.05, compared between vehicle- and IN19-administered mice (n = 4). Scale bar = 20 µm. (II) Protein extracts from the CPu of wild-type mice that were injected or not injected with MPTP/P, in the presence or absence of IN19, were subjected to Western blotting with the indicated antibodies. Relative intensities are shown in the graphs. The intensity of the signals derived from vehicle-administered mice, not injected with MPTP/P, is designated as one. Values shown are the mean ± S.D. *P<0.05, compared with mice without MPTP/P administration. ^#^P<0.05, compared between vehicle- and IN19-administered mice (n = 4).

### Reduced UPR Levels and Gene Expression in ATF6α −/− Astrocytes after MPTP/P Injections

To determine whether impaired astroglial activation was associated with reduced UPR levels in ATF6α −/− mice, expression of astrocyte-derived neurotrophic factor (BDNF), anti-oxidative genes (HO-1, xCT, and MnSOD), astrogliosis-inducing factors (LIF and IL-6), and the glutamate transporter (GLT-1) was compared with the expression of ATF6α-dependent molecular chaperones in the ER (GRP78 and ORP150) and the PERK/ATF4-dependent pro-apoptotic transcriptional factor (CHOP). Expression of the BDNF protein in ATF6α −/− mice was 62% and 68% of that in wild-type mice in the control condition and after the 3^rd^ MPTP/P injection, respectively (Fig. 4 A I). Similarly, expression of the GRP78 protein in ATF6α −/− mice was 60% and 55% of that in wild-type mice in the control condition and after the 3^rd^ MPTP/P injection, respectively (Fig. 4 A I). The expression level of BDNF, GRP78, and ORP150 mRNA in ATF6α −/− mice was 71%, 77% and 79% of that in wild-type mice in the control condition, and 150%, 74%, and 83% at twenty-four hours after the 1^st^ MPTP/P injection (Fig. 4 A II). These results suggest that the deletion of the ATF6α gene may suppress BDNF expression by transcriptional (as in the control condition) and translational/posttranslational (as in the MPTP/P-injected conditions) mechanisms.

Regarding the anti-oxidative genes, expression of the PERK/ATF4-dependent genes, HO-1 and xCT, was lower in ATF6α −/− mice than wild-type mice, at both the protein and mRNA level after MPTP/P injection, although they were not significantly different in the control conditions (Fig. 4 B I, II). Similar results were obtained with CHOP (Fig. 4 B I, II). In contrast, expression of the PERK/ATF4-independent anti-oxidative gene MnSOD was not significantly different between wild-type and ATF6α −/− mice, either in the control or after MPTP/P injection (data not shown).

Several cytokines and growth factors secreted from damaged neurons or other cells in the brain can induce astroglial activation [18]. Therefore, we examined the expression level of these genes in wild-type and ATF6α −/− mice. LIF and IL-6 expression was significantly enhanced, both at the protein (Fig. 4 C I, II) and mRNA (Fig. 4 C III) level, after MPTP/P injection in wild-type mice, but not in ATF6α −/− mice. Importantly, increased LIF and IL-6 expression in wild-type mice preceded GFAP upregulation (Fig. 4 C I). Although reduced expression of LIF and IL-6 is likely not associated with reduced GRP78 or CHOP expression in ATF6α −/− mice, these results suggest that ATF6α may transcriptionally regulate the expression of astrogliosis-inducing factors after MPTP/P injection.

Consistent with the immunohistochemical results, GLT-1 expression was not significantly different between wild-type and ATF6α −/− mice in the control condition or after MPTP/P injection (Fig. S2 D).

### UPR Activation and Neuroprotection by IN19

We previously reported that IN19 activated the UPR and protected dopaminergic neurons against acute MPTP administration [11]. In this study, we evaluated the neuroprotective property of IN19 in the chronic MPTP/P injection model. Immunohistochemical analyses revealed that IN19 administration (8×IN19 administration p.o./2 weeks) upregulated the expression of ORP150 (Fig. 5 A I, II) and GRP78 (Fig. S3 A) in TH-positive dopaminergic neurons and GFAP-positive astrocytes without reducing the number of TH-positive neurons or the intensity of TH. RT-PCR analyses revealed enhanced activation of ATFα and PERK/ATF4 pathways, but not of the Ire1/XBP1 pathway, in IN19-administrated mice (Fig. 5 B I, II, III). Unlike MPTP/P administration, IN19 administration did not upregulate GFAP or Iba1 (Fig. 5 B IV), suggesting that the effect of IN19 on UPR was not mediated by general neuronal damage. IN19 also enhanced eIF2α phosphorylation in dopaminergic neurons (Fig. S3 B), as previously described [11]. Next, we assessed the neuroprotective property of IN19 after MPTP/P injections. When mice were given IN19 (10 mg/kg, p.o. in saline, including 10% Cremophore EL and 10% DMSO) 24 h and 2 h before MPTP/P injection, the number of TH-positive neurons in the SN and the intensity of TH in the CPu were significantly increased (Fig. 6 A). Consistently, the number of activated caspase 3-positive, TH-positive neurons (Fig. 6 B) decreased in the SN, and expression of BDNF in the CPu increased in the astrocytes of mice given IN19 after MPTP/P injection (Fig. 6 C I, II). Importantly, expression of GFAP in the CPu also mildly, but significantly, increased in mice given IN19 after MPTP/P injection (Fig. 6 C I, II), suggesting that IN19 may protect dopaminergic neurons, at least in part, through the activated astrocytes after MPTP/P administration.

## Discussion

In this study, we first demonstrated the activation of the UPR in a chronic MPTP/P injection model. Of the 3 UPR branches, the ATF6α and PERK/eIF2α/ATF4 pathways were preferentially activated after MPTP/P injections (Fig. 1 B). We also observed a trend that the PERK/eIF2α/ATF4 pathway was highly activated after the 1^st^ MPTP/P injection (8 h after injection; Fig. 1 B II), but the ATF6 pathway was activated for longer periods over the course of the MPTP/P injections (1^st^ through 5^th^ injections; Fig. 1 B I). These results are consistent with those of previous reports demonstrating differential activation between the 3 UPR branches after PD-related stresses caused by MPP^+^ or 6-OHDA in cultured cells [9], [19]. Taken together with a recent report, which demonstrated a direct link after MPP^+^ treatment between p38 MAP kinase and ATF6α [12], these findings suggest critical roles for the ATF6α and PERK/eIF2α/ATF4 pathways as defense systems against PD-related neurotoxins.

Analyses of wild-type and ATF6α −/− mice showed accelerated degeneration of the nigrostriatal neurons in ATF6α −/− mice (Fig. 2 A I, II, III) after the earlier MPTP/P injections (2^nd^ and 3^rd^ injections), but not after the later injections (6^th^ through10^th^ injections). Similarly, Ub accumulation was observed in ATF6α −/− dopaminergic neurons after the early MPTP/P injections (2^nd^ and 3^rd^ injections; Fig. 2 B I). However, Ub-positive inclusions, which were abundantly observed in ATF6α −/− mice after acute MPTP injection [12], were observed only in 29% of ATF6α −/− mice after the last injection (10^th^ injection; Fig. 2 B II). These results suggest that ATF6α may contribute to neuronal survival and protein aggregation regulation in the early stages, but not in the late stages, of PD. Regarding the cell death pathways involved in our model, increased expression of activated caspase-3 was observed in ATF6α −/− dopaminergic neurons after MPTP/P injections. However, the expression of CHOP, a mediator of ER stress-induced cell death, was reduced in ATF6α −/− mice compared with wild-type mice after MPTP/P injections (Fig. 4 A II, C II). These data suggest that the accelerated neuronal death in ATF6α −/− dopaminergic neurons after MPTP/P injection may include ER stress-induced, CHOP-independent neuronal death. This is consistent with the finding of a previous report demonstrating that null mutation of CHOP did not protect against neuronal loss in a chronic MPTP/P model [20].

Interestingly, astroglial activation was strongly suppressed, and biosynthesis of the neurotrophic factor BDNF and the anti-oxidative gene heme oxygenase-1 (HO-1) was reduced in ATF6α −/− mice after MPTP/P injections (Fig. 3 B I, II). Astrocytes are ubiquitous in the brain, and upon central nervous system insult, undergo molecular and morphological changes, referred to as reactive astrogliosis or astroglial activation [18]. Activated astrocytes enhance the neuronal survival by secreting neurotrophic factors or antioxidants, as well as by reducing glutamate levels in extracellular spaces [16], [17]. In our model, reduced BDNF expression was observed in ATF6α −/− mice and was associated with reduced GRP78 expression (Fig. 4 A, B). These results were consistent with those of our previous reports that ATF6α-dependent molecular chaperones, such as GRP78 and ORP150, promote the maturation of neurotrophic factors in the ER [21], [22]. Expression of the PERK/ATF4-dependent anti-oxidative genes HO-1 and xCT, but not the PERK/ATF4-independent anti-oxidative gene MnSOD was also reduced at both the protein and mRNA level in ATF6α −/− mice after MPTP/P injections (Fig. 4 A). These results suggest that some of PERK/ATF4-dependent anti-oxidative genes are also transcriptionally regulated by ATF6α, similar to PERK/ATF4-dependent pro-apoptotic gene CHOP [23]. Although it is currently unknown which steps in astroglial activation are impaired in ATF6α −/− mice, it is possible that extracellular signals including LIF and IL-6, from damaged neurons or other cells in the brain, are too low to promote activation in ATF6α −/− astrocytes. Alternatively, it is also possible that enhanced levels of ER stress in ATF6α −/− astrocytes compromised intracellular signals which are important for the astroglial activation, as was recently reported for hepatocytes [24].

Consistent with the results from ATF6α −/− mice, administration of the IN19 to wild-type mice enhanced UPR-target gene expression, including ORP150 and GRP78, in both nigrastriatal neurons and astrocytes, and facilitated neuronal survival after MPTP/P injection. These results are consistent with those of previous reports demonstrating that IN19 can distribute into the brain after oral administration [25], and protect cells in both the ER stress model and acute MPTP injection model [11], [25]. Although IN19 alone did not cause astrogliosis (Fig. 5 B IV), IN19 administered in the course of MPTP/P injections enhanced expression of GFAP (Fig. 6 C I, II) mildly, but significantly, suggesting that IN19 may protect dopaminergic neurons, at least in part, through the activated astrocytes after MPTP/P administration. A recent report demonstrated that Salubrinal, a compound that regulates ER stress by activating the eIF2α/ATF4 pathway, attenuated disease manifestation in the A53T α-synuclein-overexpressed PD model [26]. These results emphasize the protective role of the UPR in PD.

In conclusion, we found that the UPR branches were activated in a mouse model of chronic MPTP/P injection, and they contributed to nigrostriatal neuronal survival, at least in part, through activated astrocytes. Further studies to dissect the neuron-glial association through the UPR should provide novel therapeutic window for PD and other neurodegenerative diseases.

## Supporting Information

Figure S1 **Astrocyte and microglia activation in a mouse model of chronic MPTP/P injection.** Total RNA (1 µg) isolated from brain samples, including the ventral midbrain, after MPTP/P injections was subjected to RT-PCR with specific primers for GFAP (activated astrocytes) and Iba1 (activated microglia) as described in Fig. 1 B. The relative intensity of the bands derived from mice without MPTP/P administration is designated as one. Values shown are the mean ± S.D. *P<0.05, **P<0.01 compared with mice not administered MPTP/P (n = 4).
(TIF)

Figure S2 **Immunohistochemical analysis of wild-type and ATF6α −/− brains after MPTP/P injections.** A, Negative control experiment. Brain sections, including the SN from wild-type and ATF6α −/− mice after MPTP/P injection, were incubated with mouse anti-TH antibody, followed by incubation with both anti-rabbit Alexa Fluor 488 and Cy3-conjugated anti-mouse IgG. Scale bars = 30 µm (low mag.), 15 µm (high mag.).B, Serial photograph of activated caspase 3 in wild-type mice after MPTP/P injections. Brain sections, including the SN from wild-type mice after MPTP/P injection, were immunostained with TH and activated caspase 3 antibodies. The nuclei are stained with DAPI. Scale bar = 15 µm. C, Immunohistochemical analyses of GLT-1. Brain sections, including the CPu, from wild-type and ATF6α −/− mice after MPTP/P injections were immunostained with GLT-1 and GFAP antibodies. Nuclei were stained with DAPI. Scale bar = 30 µm. D, Western blot analyses for GLT-1. Protein extracts from brains (CPu) of wild-type and ATF6α −/− mice that were injected or not injected with MPTP/P were subjected to Western blot with the GLT-1 antibody.
(TIF)

Figure S3 **UPR activation and astrogliosis after tangeretin (IN19) administration.** GRP78 expression (A), eIF2α activation (B) in the SN. Brain sections, including the SN, from wild-type mice administered or not administered IN19 for 2 weeks (4 times/week) were immunostained with the GRP78, phosphorylated eIF2α, and TH antibodies. Arrows indicate activated (phosphorylated) eIF2α in TH-positive neurons. Scale bars = 30 µm (A), 20 µm (B).
(TIF)
