# Supplementary figures and images for: ATF6alpha Promotes Astroglial Activation and Neuronal Survival in a Chronic Mouse Model of Parkinson’s Disease
Source: PLoS One. 2012 Oct 24;7(10):e47950. doi: 10.1371/journal.pone.0047950 (PMC3480445; doi:10.1371/journal.pone.0047950)

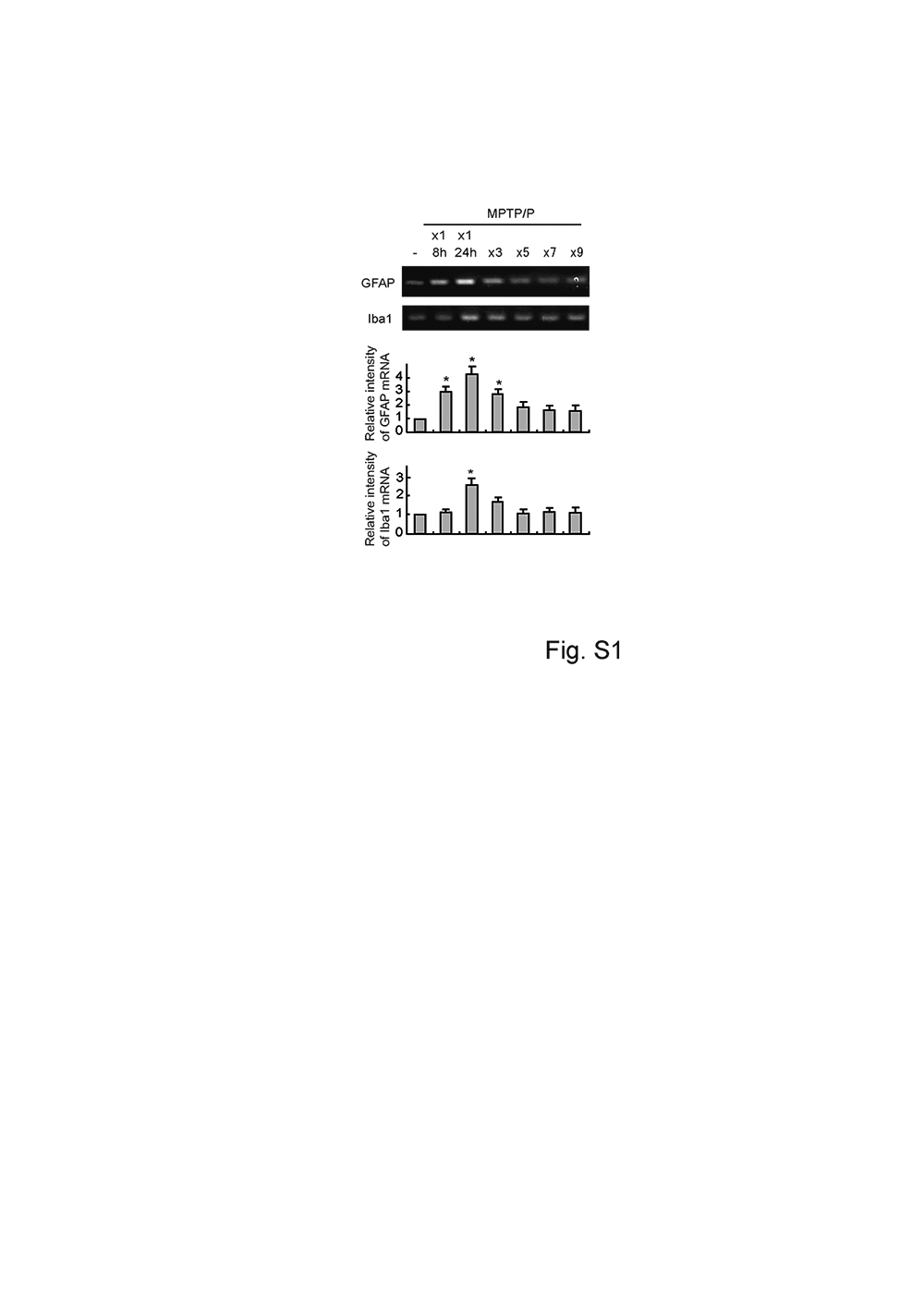

Supplement: Figure S1 — Astrocyte and microglia activation in a mouse model of chronic MPTP/P injection. Total RNA (1 µg) isolated from brain samples, including the ventral midbrain, after MPTP/P injections was subjected to RT-PCR with specific primers for GFAP (activated astrocytes) and Iba1 (activated microglia) as described in Fig. 1 B. The relative intensity of the bands derived from mice without MPTP/P administration is designated as one. Values shown are the mean ± S.D. *P<0.05, **P<0.01 compared with mice not administered MPTP/P (n = 4). (TIF) [file pone.0047950.s001.tif]

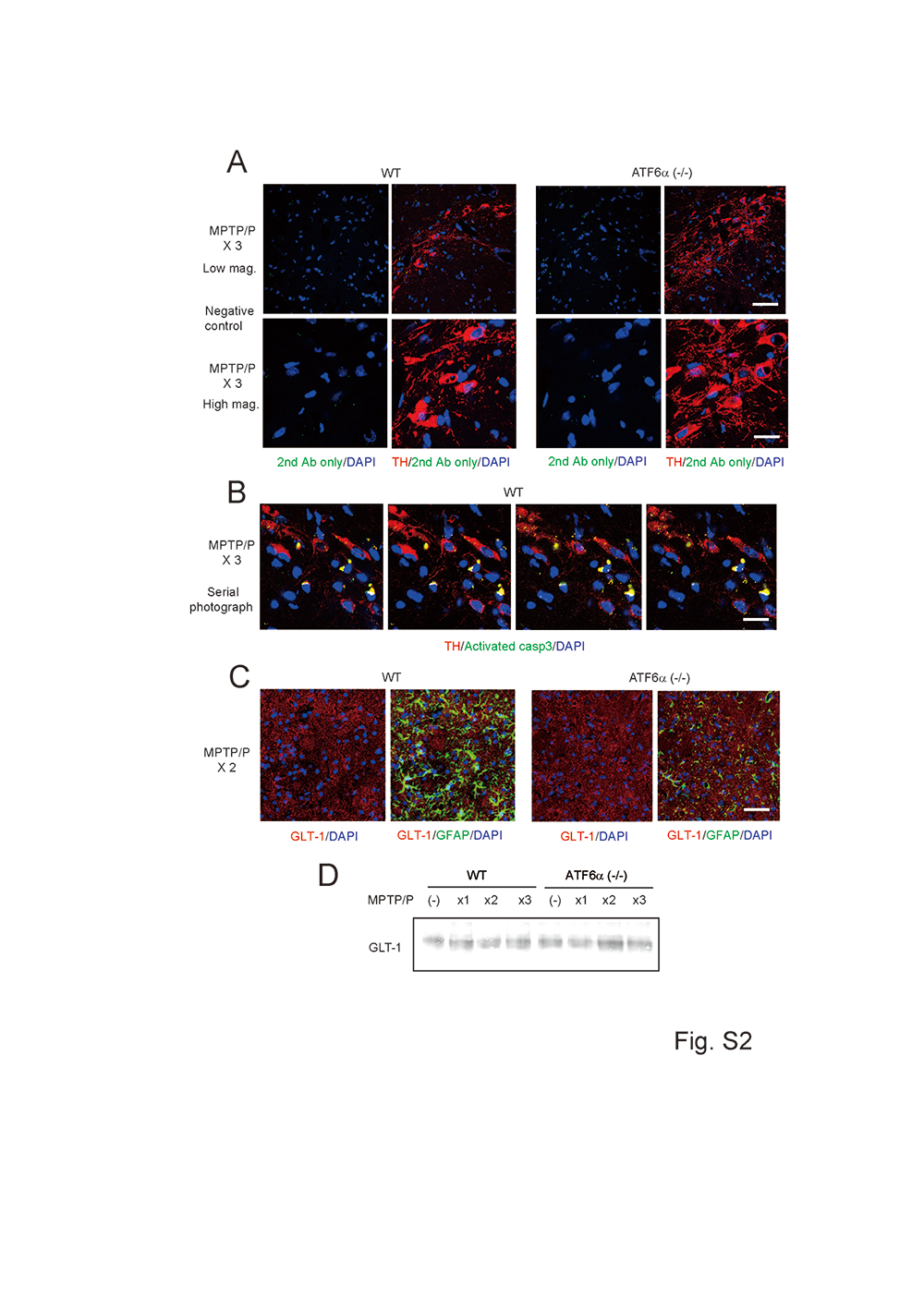

Supplement: Figure S2 — Immunohistochemical analysis of wild-type and ATF6α −/− brains after MPTP/P injections. A, Negative control experiment. Brain sections, including the SN from wild-type and ATF6α −/− mice after MPTP/P injection, were incubated with mouse anti-TH antibody, followed by incubation with both anti-rabbit Alexa Fluor 488 and Cy3-conjugated anti-mouse IgG. Scale bars = 30 µm (low mag.), 15 µm (high mag.).B, Serial photograph of activated caspase 3 in wild-type mice after MPTP/P injections. Brain sections, including the SN from wild-type mice after MPTP/P injection, were immunostained with TH and activated caspase 3 antibodies. The nuclei are stained with DAPI. Scale bar = 15 µm. C, Immunohistochemical analyses of GLT-1. Brain sections, including the CPu, from wild-type and ATF6α −/− mice after MPTP/P injections were immunostained with GLT-1 and GFAP antibodies. Nuclei were stained with DAPI. Scale bar = 30 µm. D, Western blot analyses for GLT-1. Protein extracts from brains (CPu) of wild-type and ATF6α −/− mice that were injected or not injected with MPTP/P were subjected to Western blot with the GLT-1 antibody. (TIF) [file pone.0047950.s002.tif]

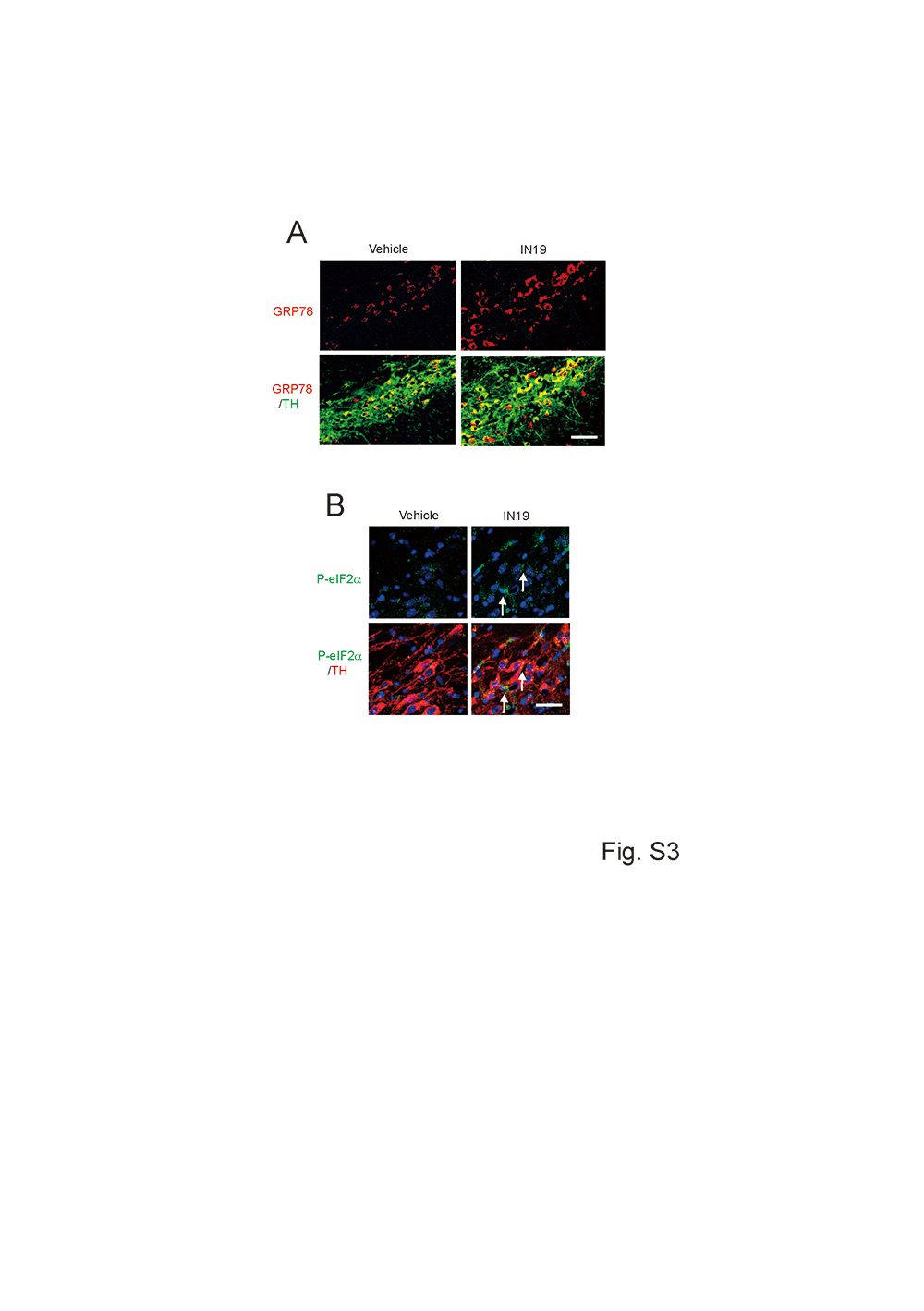

Supplement: Figure S3 — UPR activation and astrogliosis after tangeretin (IN19) administration. GRP78 expression (A), eIF2α activation (B) in the SN. Brain sections, including the SN, from wild-type mice administered or not administered IN19 for 2 weeks (4 times/week) were immunostained with the GRP78, phosphorylated eIF2α, and TH antibodies. Arrows indicate activated (phosphorylated) eIF2α in TH-positive neurons. Scale bars = 30 µm (A), 20 µm (B). (TIF) [file pone.0047950.s003.tif]
